# Drug combination discovery assisted by AI and untargeted metabolomics: pamiparib and anlotinib synergistic potentiation for ovarian cancer treatment

**DOI:** 10.3389/fphar.2025.1702014

**Published:** 2025-11-26

**Authors:** Dong Wang, Baiwang Chu, Tingjie Wen, Xueyu Wang, Ying Li, Hanning Chen, Zhao Zhang, Hua Sun

**Affiliations:** 1 Tianjin Medical University Cancer Institute and Hospital, National Clinical Research Center for Cancer, Key Laboratory of Cancer Prevention and Therapy, Tianjin’s Clinical Research Center for Cancer, Tianjin, China; 2 Tianjin Cancer Hospital Airport Hospital, Tianjin, China; 3 College of Biotechnology, Tianjin University of Science and Technology, Tianjin, China; 4 College of Mechanical Engineering, Tianjin University of Science and Technology, Tianjin, China

**Keywords:** pamiparib, anlotinib, ovarian cancer, cancer stem cell (CSC), untargeted metabolomics, artificial intelligence (AI)

## Abstract

**Background:**

The aim of this study was to investigate the mechanism of the Poly ADP-ribose polymerase (PARP) inhibitor pamiparib (PAM) and the tyrosine kinase inhibitor anlotinib (ANL), utilizing a Biological Factor Regulatory Neural Network (BFReg-NN) and metabolomics approach.

**Methods:**

Potential drug combinations were identified by integrating bioinformatics and machine learning algorithmic strategies. *In vitro*, their effects on ovarian cancer cells were detected by MTT assay, clone formation and annexin V/PI double staining, scratch assay and Transwell assay. The effect of PAM in combination with ANL was investigated in a nude mouse ovarian cancer model. The mechanism of action was investigated using an untargeted metabolomics approach. The inhibitory effect of the combination of the two drugs on stem cell activity was detected using the tumorsphere assay, limiting dilution assay and RT-qPCR, and the changes in signaling pathway protein expression after treatment with the two drugs were detected using Western blotting.

**Results:**

Predictive results confirmed the synergistic effect of the potential drug combinations, revealing that the potential mechanism of PAM combined with ANL in ovarian cancer is related to tumor stem cells. Overexpression of the PI3K/Akt signaling pathway is commonly associated with cancer recurrence and drug resistance. *In vitro*, the combination of PAM and ANL inhibited clone formation, proliferation, migration, and stemness of A2780 ovarian cells through the PI3K/Akt signaling pathway. *In vivo*, significant downregulation of p-PI3K, p-Akt, Bcl-2, and HIF1-α, and upregulation of BAX protein expression confirmed that the mechanism of action of combination therapy is related to PI3K/Akt pathway.

**Conclusion:**

The combination of PAM and ANL was more effective than monotherapy for treating ovarian cancer and holds potential to become a new therapeutic approach for ovarian cancer.

## Graphical Abstract

**Figure:**
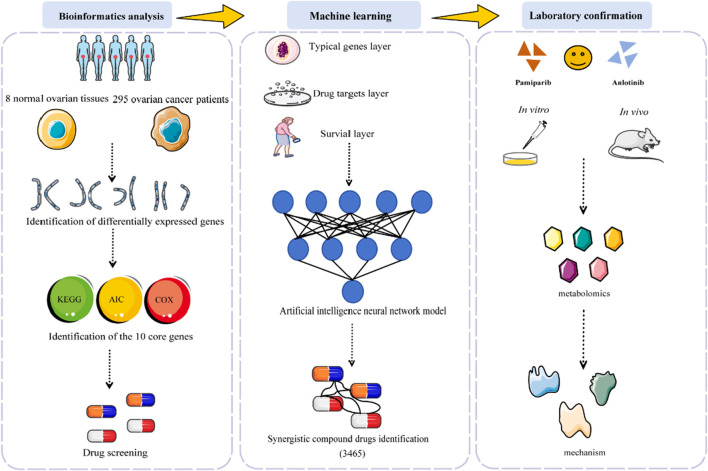

## 1 Introduction

Ovarian cancer is highly prevalent and has the highest mortality rate among gynecological malignancies worldwide (Kuroki and Guntupalli, 2020). Although conventional first-line treatments, such as tumor cytoreduction and postoperative adjuvant platinum-based combination chemotherapy, bring about a clinical remission in most patients, 70% of patients still experience relapse within 3 years (Giornelli, 2016). Patients who become tolerant to chemotherapy after relapse have a 5-year survival rate of only 29% (Lheureux et al., 2019; Guleria et al., 2020). Therefore, there is an urgent need to identify new and effective treatments to reduce the mortality rate of patients with ovarian cancer.

Artificial Intelligence (AI) has begun to accelerate its application in all areas of society, with the biomedical sector showing outstanding potential (Paul et al., 2021). Recently, an increasing number of researchers have used neural network models to predict drug combination synergies (Ouyang et al., 2024; Wang T. et al., 2024). Poly ADP-ribose polymerase (PARP) inhibitors, such as pamiparib (PAM), niraparib, olaparib, and fluzoparil, are promising agents for treating BRCA-related cancers, especially high-grade ovarian and breast cancers. PARP inhibitors cause “synthetic lethality” in BRCA mutant cells by blocking replication forks in homologous recombination-defective (HRD) cells (Desai et al., 2022). Anlotinib (ANL) is a multitargeted tyrosine kinase inhibitor with antiangiogenic activity. It induces microenvironmental hypoxia (Liu et al., 2022), which inhibits HRD in human cells. Hypoxic cancer cells that lack HRD can become more sensitive to PARP inhibitors (Hegan et al., 2010). Hypoxia can inhibit BRCA1 expression, leading to functional BRCA1 inactivation without genetic mutations (Bindra et al., 2005). Furthermore, PARP inhibitors can also have antiangiogenic effects (Tentori et al., 2007). In combination with neural network modelling, we found that the combination of PAM and ANL could produce a synergistic effect. This may provide a new strategy for the treatment of ovarian cancer patients without BRCA gene mutations but with normal HRD function.

Metabolomics is an emerging methodology for identifying changes in metabolic profiles in a systems biology approach, which employs advanced analytical chemistry techniques to determine the dynamics of metabolites in the body to reflect the overall metabolic levels, physiology, and pathology of an organism (Shi et al., 2023; Wang S. et al., 2024). Non-targeted metabolomics can capture a wide range of metabolite information, unearth potential biomarkers and metabolic pathways, and provide opportunities for analyzing the mechanism of action of drug combinations (Mastrangelo et al., 2014; Qiu et al., 2023).

Hence, in this study, we investigated and demonstrated the synergistic interaction of two anticancer drugs, PAM and ANL, assisted by Biological Factor Regulatory Neural Network (BFReg-NN) and non-targeted metabolomics.

## 2 Materials and methods

### 2.1 Materials

Ovarian cell lines SK-OV3 and A2780 were purchased from Shanghai Fuheng Biological Co. (Shanghai, China), and OV-CAR5 was stored in the lab. Roswell Park Memorial Institute (RPMI) 1640, high-glucose Dulbecco’s modified Eagle medium (DMEM), 0.25% trypsin solution, and penicillin-streptomycin solution were purchased from Grand Island Biological Co., Ltd. (NY, United States). Dulbecco’s modified Eagle medium nutrient mixture F-12 (DMEM/F-12, 1:1) was purchased from Thermo-Fisher Biochemical Products Co., Ltd. (Beijing, China). Fetal bovine serum (FBS) was purchased from Lanzhou Minhai Biotechnology Co., Ltd. (Gansu, China). Dimethyl sulfoxide (DMSO), thiazolyl blue (MTT), crystal violet solution, phospholipid-binding protein (ANNEXIN V)-FITC/propidium iodide (PI) apoptosis detection kit, and BCA protein concentration measurement kit were purchased from Solarbio Life Sciences (Beijing, China). FGF basic/bFGF and EGF were purchased from MedChemexpress Biotechnology Co., Ltd. (New Jersey, USA). The BALB/C nude mice were purchased from Beijing Vital River Laboratory Animal Technology Co., Ltd. (Beijing, China) [License SCXK (Jing) 2021–0006]. Ultrapure water was purchased from Watson’s Co., Ltd. (Shanghai, China). Acetonitrile, methanol, formic acid, and ammonium formate were purchased from Aladdin (Shanghai, China), and were LC-MS grade pure. PAM (pamiparib capsules) were provided by BeiGene, Inc. (Suzhou, China). ANL (anlotinib hydrochloride capsules) were provided by Chia Tai Tianqing Pharmaceutical Group Co., Ltd. (Jiangsu, China). Alanine aminotransferase (ALT) and aspartate aminotransferase (AST) assay kits were purchased from Nanjing Jiancheng Bioengineering Institute (Jiangsu, China). The following antibodies, including phosphatidylinositol kinase (PI3K), p-PI3K, serine/threonine kinase (Akt), p-Akt, neural calcium mucin (N-cadherin), epithelial calcium adhesion protein (E-cadherin), B-lymphoblastoma-2 gene (Bcl-2), BCL2-associated X (Bax), SRY box-2 (SOX2), aldehyde dehydrogenase 1A1 (ALDH1A1), hypoxia-inducible factor 1 subunit alpha (HIF1-α), Nuclear factor erythroid 2-related factor 2 (Nrf2) and microtubule protein (Tubulin) were purchased from Chengdu Zheng neng Biological Co., Ltd. (Sichuan, China). Polyvinylidene difluoride membranes (PVDF) were purchased from Merck Millipore Ltd. (Tullagreen, Carrigtwohill, Co. Cork, Ireland).

### 2.2 Drug combination screening

#### 2.2.1 Data acquisition

AffyU133a (n = 593) and gene expression subtyping (n = 308) TCGA Hub files were downloaded from the UCSC Xena (UCSCX) database (https://xena.ucsc.edu/). Gene expression matrices and subtyping data of 8 normal ovarian tissues and 295 ovarian cancer tissues of patients were obtained and analyzed using the “limma” R software package. Given 1168 differentially expressed genes, we used the Kyoto Encyclopedia of Genes and Genomes (KEGG) to enrichment analyze using the Metascape website (https://metascape.org/gp/index.html#/main/step1). Heatmap data were produced using the Microbiome website (http://www.bioinformatics.com.cn/). The DGldb database (https://dgidb.org/) was utilized to find drugs based on genes. Pubchem database (https://pubchem.ncbi.nlm.nih.gov/) and SwissTargetPrediction website (www.swisstargetprediction.ch/) were used to predict the possible targets of drugs.

#### 2.2.2 Artificial intelligence–based drug combination prediction (BFReg-NN)

To ensure interpretability and reproducibility, the Biological Factor Regulatory Neural Network (BFReg-NN) was constructed following the architecture proposed by Ouyang et al. (2024). BFReg-NN defines its neural-network structure according to biological hierarchy. $L=\left[Gene,Protein,Pathway,Survival\right]$ .

For each level *I*, the intra-level regulatory relations are represented by a binary adjacency matrix $A_{l}=\left\{A_{Gene},A_{Protein},A_{Pathway},...\right\}$ , derived respectively from Gene Regulatory Network (GRN), Protein–Protein Interaction (PPI), and pathway ontology (GO/KEGG). Inter-level mappings are described by a set of matrices $M=\left\{M_{1},M_{2},...,M_{L-1}\right\}$ ,where $M_{1}$ maps genes to proteins, $M_{2}$ maps proteins to pathways, and the combination of *A* and *M* defines the overall topology of the neural network, including nodes and their connections.

Each biological factor *i* at level *I* encoded by an embedding $H_{i}^{l,0}=emb\left(x_{i}\right)$ . Message-passing within each level follows a graph-neural-network (GNN) rule: ${\hat{H}}_{i}^{l}=\text{update}\left(\sum_{j\in A_{l}\left(i\right)}message\left(H_{i}^{l},H_{j}^{l}\right),H_{i}^{l}\right)$ , where neighboring nodes $A_{l}\left(i\right)$ transmit regulatory information. Cross-level propagation then updates ${\hat{H}}_{i}^{l+1}=\sigma \left(\left(M_{1}⊙W^{l}\right){\hat{H}}_{i}^{l}+b^{l}\right)$ , in which $W^{l}$ and $b^{l}$ are trainable parameters and ⊙ denotes element-wise multiplication ensuring absent relations are masked.

To explore latent biological regulation, BFReg-NN introduces a learnable adjacency matrix $A_{l}^{\prime }\left(i\right)=\left\{\begin{array}{l} \beta_{ij}^{l},\begin{array}{ccc} & & if\;universal\;regulation\;exists\;in\;A_{l} \end{array}\\ \alpha \beta_{ij}^{l},\begin{array}{ccc} & & if\;interaction\;is\;absent\;in\;A_{l} \end{array} \end{array}\right.$ , where α=0.005 serves as a small penalty for uncertain edges, and $\beta_{ij}^{l}$ is earned via a Multilayer Perceptron (MLP) on embeddings $H_{i}^{l},H_{j}^{l}$ .This mechanism enables BFReg-NN to integrate known biological knowledge while discovering new potential associations.

Each drug is represented by its target-gene set extracted from DGIdb, and each target gene carries normalized expression values from TCGA ovarian-cancer transcriptomes (TPM scale). The final input tensor combines drug–target adjacency matrices $\left(A_{\text{drug}},A_{\text{target}}\right)$ and corresponding gene-expression embeddings.

The network was trained on 80% of all possible drug combinations and validated on the remaining 20%. Optimization used Adam (learning rate 1 × 10^−4^, batch size 32, 200 epochs) with early-stopping based on validation loss. The Cox proportional hazards loss was adopted $L_{\text{Cox}}=-\sum_{i}\left(H_{i}-\log\sum_{j\in R_{i}}e^{H_{j}}\right)$ and model performance was evaluated by the concordance index (c-index), where values >0.9 indicate strong consistency between predicted and observed survival outcomes.

For each drug pair, the trained BFReg-NN predicts recurrence-free survival (RFS) of ovarian-cancer patients. Higher predicted RFS correlation between two drugs implies stronger therapeutic synergy. Integrated-Gradient analysis was used to interpret model weights and identify key genes mediating the synergy between PAM and ANL.

### 2.3 Cell culture

SK-OV3 cells were cultured in RPMI 1640 medium, while OV-CAR5 and A2780 cells were cultured in DMEM high-glucose medium. The complete medium comprised 1% penicillin-streptomycin solution and 10% FBS. Cells were cultured at 37 °C in a 5% CO_2_ incubator.

### 2.4 MTT assay

Ovarian cancer cells were seeded in 96-well plates at a density of 5 × 10^4^ cells/well and allowed to adhere. The cells were treated with 0.5 μL of varying concentrations of the tested drug or DMSO for 48 h. After treatment, the cells were incubated with 20 μL MTT solution for 4 h at 37 °C. Following the incubation period, the medium was aspirated, and each well was filled with 100 μL DMSO and incubated for 10 min. Finally, the absorbance of each well was measured at 492 and 630 nm using a microplate reader (Thermo-Fisher Scientific, Shanghai, China).

### 2.5 Determination of drug interaction

Drug interaction assays were conducted to investigate the synergistic effects of PAM and ANL co-administration using the Chou-Talalay method (Chou, 2010). Ovarian cancer cells were treated with 0.5 μL of varying concentrations of PAM (final doses of 8, 4, 2, 1, 0.5, or 0.25 μM) and ANL (final doses of 8, 4, 2, 1, 0.5, or 0.25 μM), and their optimal dosages were selected based on their respective semi-inhibitory concentration values (IC_50_). Cell viability was assessed after 24 h of treatment using the MTT assay. The effect categories were determined by calculating the Combination Index (CI) using calculation software (version 1.0; CompuSyn, Paramus, NJ, United States). Antagonistic, additive, and synergistic effects are expressed as CI > 1, CI = 1, and CI < 1, respectively.

### 2.6 Colony formation assay

Cells were seeded in 6-well plates at a density of 1000 cells/well. After adhering to the plates, cells were treated with 10 μL of 2 μM PAM, 2 μM ANL, or 2 μM PAM +2 μM ANL. The cells in the control group were treated with equal amounts of DMSO. The medium was replaced with fresh DMEM supplemented with 10% FBS and 1% penicillin/streptomycin every 3 days and treated as described above. After 12 days of incubation, cells were fixed with 4% paraformaldehyde, washed twice with PBS, and stained with 0.1% crystal violet. The optimal number of clones was determined.

### 2.7 Wound healing assay

Ovarian cancer A2780 cells were uniformly inoculated in a 6-well plate at a density of 3 × 10^5^ cells/well to allow adhesion. Cells were incubated with 10 μL of DMSO (control) or tested drug or a combination thereof at 37 °C and 5% CO_2_. Scratches were made on the monolayers when the cells were nearly confluent, and cell migration was recorded and photographed at 0, 24, and 48 h under a microscope (Olympus, Tokyo, Japan). The Situ Image Acquisition System software was used to analyze the changes in wound width.

### 2.8 Transwell assay

A suspension of 1 × 10^5^ A2780 cells in 200 μL of serum-free medium was added to the upper chamber of the Transwell inserts. The suspension contained 1 μL of 2 μM PAM, 2 μM ANL,2 μM PAM + 2 μM ANL, or DMSO (control). The medium in the lower chamber was supplemented with 600 μL medium and 20% FBS. After incubation at 37 °C for 24 h, cells that crossed the membrane were fixed in 4% formaldehyde at 25 °C for 20 min. They were then washed twice with PBS and soaked in 0.1% crystal violet solution for 30 min. Finally, the cells were counted using an inverted microscope.

### 2.9 Determination of apoptosis

Ovarian cancer cells were inoculated uniformly into 6-well plates at a density of 5 × 10^5^ cells/well. After allowing the cells to adhere, they were incubated with either 10 μL of DMSO, or 2 μM PAM, 2 μM ANL, or a combination thereof for 48 h. The medium was then aspirated, and the cells were washed three times with pre-cooled PBS. Subsequently, the cells were digested with trypsin without EDTA. Cells were incubated with membrane-linked protein V-FITC dye for 5 min at 4 °C in the dark, followed by incubation with PI for an additional 5 min at 4 °C in the dark. Apoptotic cells were detected using flow cytometry.

### 2.10 Tumor sphere assay

Cells in the logarithmic growth phase were inoculated into well plates at a density of 1 × 10^5^ cells/well. After incubation for 24 h, 10 μL of DMSO, or 0.5 μM of the tested drug, or a combination thereof, was added to the corresponding wells, and the cells were treated for 48 h. The cells were digested with trypsin and centrifuged in normal medium, followed by secondary centrifugation with PBS. The supernatant was discarded, and each group of cells was resuspended in a serum-free tumor sphere-forming medium (20 ng/mL EGF, 20 ng/mL bFGF, and 2% B27 in DMEM:F12). Cells were counted in each group by inoculating them at a density of 1000 cells per well in a low-adherence 24-well plate. The cells were then carefully dispersed to ensure a single distribution without clustering. Three replicate wells were used for each concentration gradient. The plates were transferred to a cell culture incubator for incubation, photographed, and counted under a microscope (Olympus, Tokyo, Japan) after 7 days.

### 2.11 Stem cell spheroidal limiting dilution assay

Cells in the logarithmic growth phase were inoculated at a density of 1 × 10^5^ cells/well and cultured in an incubator for 24 h. 10 μL of DMSO, or 0.5 μM of the tested drug, or a combination thereof was added to the corresponding wells, and the cells were treated for 48 h. The cells were digested with trypsin, centrifuged using a normal medium, and then washed twice with PBS. The supernatant was discarded, and the cells were resuspended in a serum-free tumor sphere-forming medium for tumor sphere formation. The cells were counted and inoculated into 96-well plates. Each sample was divided into five groups based on the number of cells (200, 100, 50, and 25) in a decreasing gradient, and each group was established with 10 replicate wells. After 9 days of incubation, the cells were counted. Data were analyzed using the Limit Analysis online analysis program (https://bioinf.wehi.edu.au/software/elda/).

### 2.12 Quantitative real-time PCR (qRT-PCR)

A2780 cells were grown logarithmically and inoculated with 3 × 10^5^ cells in 6-well plates. After 24 h, 10 μL of DMSO, or 400 μM of the tested drug, or a combination thereof was added and allowed to react for 48 h. RNA was extracted using the Biozol total RNA extraction reagent (BioFlux, Beijing, China) according to the manufacturer’s instructions and then subjected to reverse transcription with the BioRT Master HiSensi cDNA First Strand Synthesis kit (BioFlux, Beijing, China). Cancer stem cells (CSC) primers (ALDH1A1, SOX2) and GAPDH (control) were synthesized by Tsingke Biotechnology Co., Ltd. (Beijing, China). The primer sequences are as follows:

ALDH1A1: 5′-ACA​GGA​TCA​ACA​GAG​GTT​GGC-3' (Forward), 5′-ATG​CAA​GGG​CTC​TTT​CCT​CC-3' (Reverse)

SOX2: 5′-GCC​GAG​TGG​AAA​CTT​TTG​TCG-3' (Forward), 5′-GGC​AGC​GTG​TAC​TTA​TCC​TTC​T-3' (Reverse)

GAPDH: 5′-GGA​GCG​AGA​TCC​CTC​CAA​AAT-3' (Forward), 5′-GGC​TGT​TGT​CAT​ACT​TCT​CAT​GG-3' (Reverse)

The values were obtained as the threshold cycle for each gene and normalized to the internal reference gene (GAPDH). The relative changes in each gene of cell lines were calculated using the 2^−ΔΔCT^ method.

### 2.13 Determination of antitumor activity *in vivo*

Female BALB/c mice, aged 4 weeks and weighing 15–20 g, were maintained under specific pathogen-free conditions. All animal experiments were conducted in accordance with the guidelines of the Animal Experimentation Center of the Tianjin University of Science and Technology and strictly compliant with the Chinese Law on the Use and Care of Laboratory Animals. The Animal Ethics Committee of the Tianjin University of Science and Technology approved the experimental protocol. To construct a xenograft tumor model, each mouse received a subcutaneous injection of 5 × 10^7^ A2780 cells near the right axilla. The size and weight of the tumors were measured every alternate day. The maximum longitudinal and transverse diameters of each tumor were measured using calipers. The total tumor volume was calculated as (length × width^2^)/2 When the tumor volume reached 100–150 mm^3^, the mice were randomly divided into the following groups and administered the indicated treatments daily: saline (control group), PAM (15 mg/kg), ANL (1.5 mg/kg), or PAM (10 mg/kg) + ANL (1 mg/kg). Drug administration was performed daily by gavage for 16 consecutive days. Animal survival was monitored and documented daily to assess potential treatment-associated mortality. After sacrifice, the tumor growth inhibition (TGI) rate was calculated using the formula: TGI (%) = (1- tumor weight of the treatment group/tumor weight of the control group) × 100%.

### 2.14 Non-targeted metabolomics

For non-targeted metabolomics analysis, the number of cells was adjusted to 1 × 10^7^ after centrifugation. 100 mg of tumor samples were weighed, and 100 μL of tumor samples were homogenized with a 4-fold amount of water. Then, 400 μL of ice-cold methanol:acetonitrile (1:1, v/v) was added, vortexed, mixed, and sonicated in an ice bath for 15 min. The mixture was then centrifuged at 4 °C, 14000 rpm by centrifuge (5804R, Eppendorf, Germany) for 20 min, and the supernatant was taken, nitrogen-blown, and re-solubilized by adding 100 μL of 75% methanol. It was vortexed, mixed, and centrifuged again, and the supernatant was taken. An Acquity H-Class UPLC ultra-high performance liquid chromatography system (Milford, MA, USA) equipped with an ACQUITYPremier HSS T3 column was used. The mass spectrometry analysis was performed on a Waters Xevo TQ-S mass spectrometry system coupled to a Waters Xevo TQ-S instrument (Milford, MA, USA) for data acquisition. Results were analyzed using MetaboAnalyst 6.0 (https://www.metaboanalyst.ca/) and the Venn diagram was plotted using https://www.bioinformatics.com.cn (last accessed on 19 December 2024), an online platform for data analysis and visualization.

### 2.15 Western blotting

Total proteins were extracted from A2780 cells or tumor tissue, and the protein concentrations were determined by using a BCA kit. The protein samples were separated by sodium dodecyl sulfate-polyacrylamide gel electrophoresis (SDS-PAGE) and transferred to PVDF membranes. The membranes were incubated with the corresponding primary and secondary antibodies. Then, the membranes were scanned using the Odyssey Western blotting system (Amersham Pharmacia Biotech). ImageJ software was used for the gray analysis of protein bands.

### 2.16 Immunohistochemical staining

Paraffin-embedded tumor tissue sections were deparaffinized and rehydrated, followed by heating in antigen retrieval solution (Beyotime Biotechnology, Shanghai, China) at 95 °C for 1 h. After blocking with 3% bovine serum albumin (BSA), the sections were incubated with the primary antibody against ALDH1A1 (R22713, dilution ratio: 1:20, Chengdu Zhengneng Biotechnology, Chengdu, China) overnight at 4 °C. On the following day, the tissues were incubated with SignalStain Boost IHC Detection Reagent (Cell Signaling Technology, MA, USA) at room temperature for 1 h. Subsequently, the sections were washed three times with phosphate-buffered saline (PBS) and then stained with 3,3′-diaminobenzidine (DAB) peroxidase substrate (P0203, Beyotime Biotechnology, Shanghai, China). Quantitative analysis was performed using ImageJ software.

### 2.17 Statistical analysis

All data are expressed as means ± the standard deviation (SD) or means ± the standard error of the mean (SEM). Results were analyzed by one-way analysis of variance (ANOVA) or two-tailed independent samples t-tests. All data were checked for normality and homogeneity of variance before testing for treatment differences. Significant differences were determined by the least significant difference (LSD) test using GraphPad Prism 8.0 and IBM SPSS Statistics 25. Statistical significance was considered at *p* < 0.05.

## 3 Results

### 3.1 Determination of PAM and ANL combinations

**FIGURE 1:**
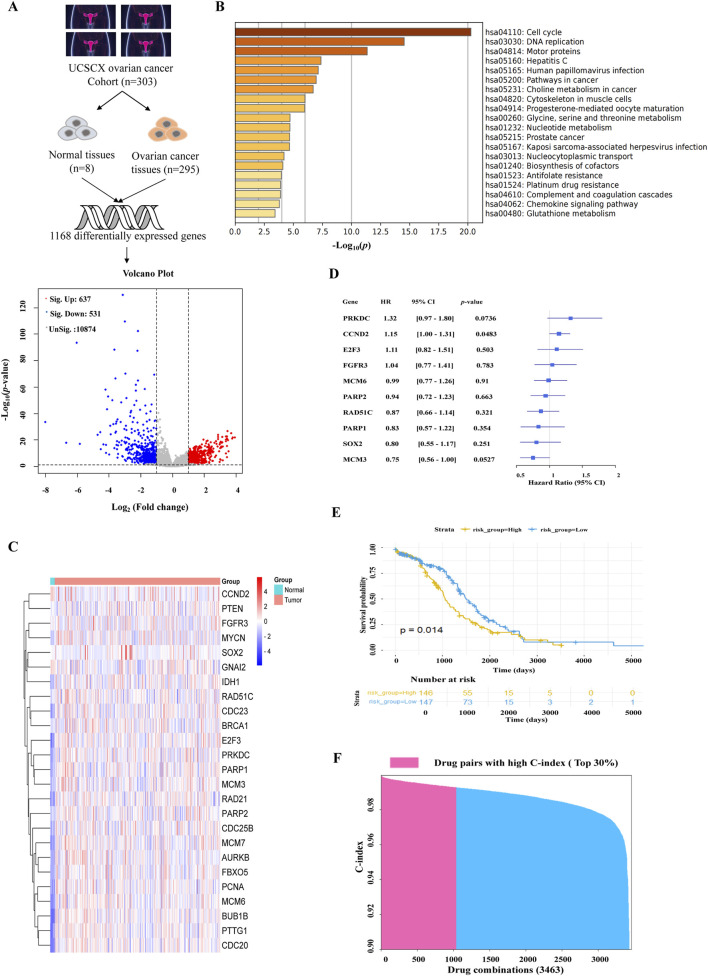
AI assisted drug combination prediction. **(A)** Differentially expressed genes were analyzed by volcano plot. **(B)** KEGG enrichment analysis of 25 typical genes. **(C)** Heat map of 25 differentially expressed genes. **(D)** Forest plot of distribution of risk ratios for typical genes. **(E)** The Kaplan-Meier overall survival curves of two clusters distinguished by gene strata (risk = high vs. risk = low: *p* = 0.014). Data are analyzed with Log-rank. **(F)** Ranked histogram of BFReg-NN predicting the correlation of drug combination with RFS in ovarian cancer.

We analyzed the gene expression matri and obtained a total of 1168 differentially expressed genes, of which 637 were upregulated and 531 were downregulated (Figure 1A). Using the Metascape website for KEGG analysis, we found that cell cycle signaling pathways were significantly enriched (Figure 1B). A total of 25 genes closely related to ovarian cancer were summarized by KEGG enrichment and previous literature (Busschots et al., 2015; Wen et al., 2017; Zhao et al., 2021; Zuo et al., 2021). Data visualization of the expression of the 25 genes showed the upregulation of the genes in tumor patients (Figure 1C). Using Cox proportional hazards model and Akaike information criterion (AIC), we finally identified 10 genes as ovarian cancer patients’ core genes, which significantly predicted recurrence-free survival (RFS) in ovarian cancer patients. In addition, the risk ratios of these 10 genes showed that *PRKDC* increased the risk of recurrence-free survival in ovarian cancer patients, while *MCM3* significantly decreased the risk (Figure 1D). We predicted Kaplan-Meier curves for ovarian cancer RFS based on the high- and low-risk profiles of these 10 genes, emphasizing their significant influence (*p* = 0.014) on the survival profile of ovarian cancer patients (Figure 1E). Based on above genes, 95 drugs were identified from the DGldb database. Furthermore, pubchem database and SwissTargetPrediction website was used to predict their potential targets. A total of 3463 drug combinations were obtained using the BFReg-NN model. The top 30% of these combinations were then screened based on the C-index, and ultimately, PAM combined with ANL was selected for further experimental validation (Figure 1F).

### 3.2 PAM and ANL combination ratio screening

**FIGURE 2:**
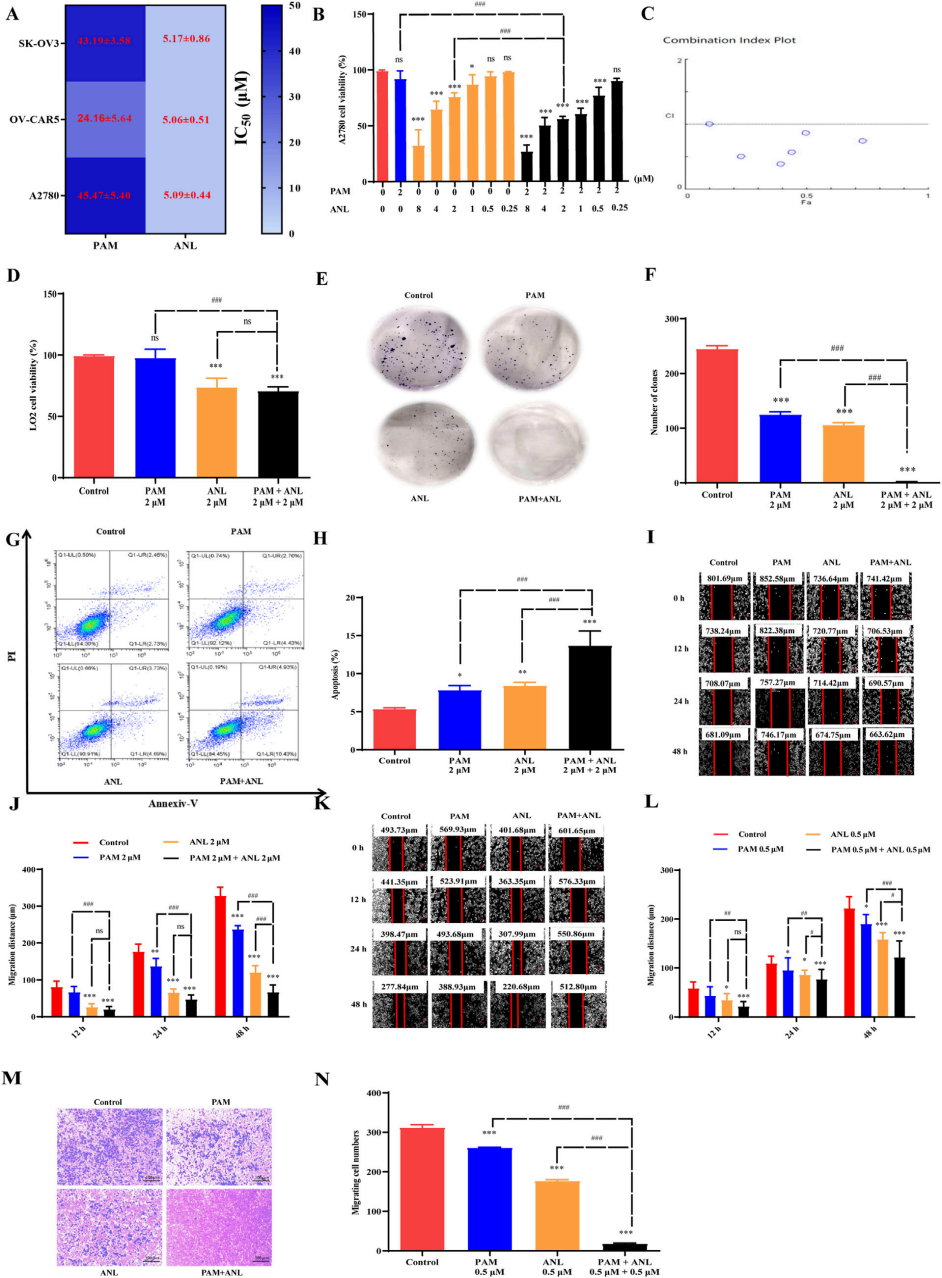
*In vitro* experimental validation of combination of PAM and ANL. **(A)** IC_50_ values of PAM and ANL at 48 h in SK-OV3, OV-CAR5, and A2780 ovarian cancer cells. **(B)** The cell survival rate of cells with or without 2 μM PAM and ANL (8, 4, 2, 1, 0.5, 0.25 μM). **(C)** CI plot of PAM combined with ANL using CompuSyn software, where Fa is the inhibition rate. CI < 1 indicates a synergistic effect, CI = 1 indicates an additive effect, and CI > 1 indicates an antagonistic effect. **(D)** Survival of normal human hepatocytes LO2 cells treated with PAM or ANL alone, or in combination. *p < 0.05, ***p < 0.001 compared with the control (DMSO) group. **(E,F)** Clone formation inhibition with PAM, ANL, or their combination. **(G,H)** The percentage of apoptotic cells was determined by flow cytometry after treatment with PAM, ANL, and their combination. **(I,J)** The results of the wound healing test showed the migration ability of cells treated with DMSO (control), PAM (2 μM), ANL (2 μM), and PAM (2 μM) + ANL (2 μM). **(K,L)** The migration ability of cells treated with DMSO (control), PAM (0.5 μM), ANL (0.5 μM), and PAM (0.5 μM) + ANL (0.5 μM). **(M,N)** Transwell assay results show the migration ability of cells treated with DMSO (control), PAM (0.5 μM), ANL (0.5 μM), and PAM (0.5 μM) + ANL (0.5 μM). All data are representative of three independent experiments, and the values are expressed as the mean ± SD. *p < 0.05, **p < 0.01, ***p < 0.001 compared with the control (DMSO) group. ^#^ *p <* 0.05, ^##^ *p <* 0.01, ^###^ *p <* 0.001 PAM or ANL alone compared with their combination.

Figure 2A shows that among the three ovarian cancer cell lines, A2780 cells were the least sensitive to PAM, with an IC_50_ value of 45.47 μM. Therefore, A2780 cells treated with PAM and ANL were selected for follow-up studies. To investigate the combined effect of PAM and ANL on A2780 cells, we used a fixed-dose and fixed-ratio screening method based on their IC_50_ values. The screening dosing ratios of PAM and ANL were determined as shown in the Supplementary Material (Supplementary Figure S1; Supplementary Table S1). When the PAM concentration was 2 μM and ANL concentrations were 8, 4, 2, 1, 0.5, and 0.25 μM, the combined action on A2780 cells showed a strong synergistic effect (CI < 1) (Figures 2B,C; Supplementary Table S1). The A2780 cell viability rates were 92.24% and 75.86% after 48 h of treatment with 2 μM of PAM and 2 μM of ANL, respectively. Notably, the combination of PAM (2 μM) and ANL (2 μM) resulted in a significantly reduced survival rate (55.94%) in A2780 cells compared with rates in the monotherapy groups (*p* < 0.001), demonstrating a good synergistic effect (CI = 0.57). For the next experiment, we used PAM combined with ANL at a concentration of 2 μM for both drugs.

To test the selectivity of PAM combined with ANL in inhibiting cell viability, normal human hepatocyte LO2 cells were treated with 2 μM PAM and 2 μM ANL. After 48 h, the cell survival rate was 71.0% (Figure 2D), which was higher than that of the A2780 cells treated with the same combination, indicating that PAM combined with ANL was less toxic to normal cells. Therefore, the combination of PAM and ANL did not exhibit superimposed toxicity in normal cells, suggesting good drug-forming properties and providing a reasonable basis for subsequent experiments.

### 3.3 PAM/ANL inhibits cell survival

To investigate the long-term effectiveness and efficiency of PAM combined with ANL in inhibiting cell viability, we examined their colony inhibition ability using the clone formation assay. When PAM monotherapy was used, the number of clones obtained was 125. When ANL alone was used, the number of clones was 106 (Figures 2E,F). However, when the two drugs were used in combination, only two clones were formed. The results of clone formation experiments showed that the combination treatment had a more significant inhibitory effect on the long-term proliferative ability of A2780 cells than monotherapy.

The apoptotic effect of the PAM and ANL combination was further studied using the annexin V-FITC and PI double staining method. The flow cytometry result showed an approximately 2-fold increase in the apoptosis rate of PAM + ANL-treated cells compared to that in the monotherapy group (Figures 2G,H). The combination treatment induced more apoptosis in A2780 cells than in the single-drug group, indicating a positive synergistic effect of the two drugs in inducing apoptosis.

### 3.4 PAM/ANL inhibits cell migration

To investigate whether this combination inhibits A2780 cell migration, a scratch assay was used to detect the lateral migration ability of the cells. The combination of PAM (2 μM) and ANL (2 μM) had a significantly inhibitory effect on migration ability compared to that in the single-drug groups (Figures 2I,J). Specifically, the migration distance was 237.34 μm for PAM, 120.03 μm for ANL, and 66.93 μm for the combination treatment at 48 h. Interestingly, the combination of PAM (0.5 μM) and ANL (0.5 μM) also significantly inhibited cell migration (Figures 2K,L). The effect of the drug combination on longitudinal migration ability was evaluated using a Transwell assay. The combination treatment significantly inhibited longitudinal migration at a concentration of 0.5 μM (Figures 2M,N). These results demonstrated that the combination of PAM and ANL significantly inhibits lateral and longitudinal cell migration even at lower concentrations, which suggests an inhibitory effect of the PAM/ANL combination on ovarian cancer cell stemness.

### 3.5 Determination of antitumor activity of PAM + ANL *in vivo*

**FIGURE 3:**
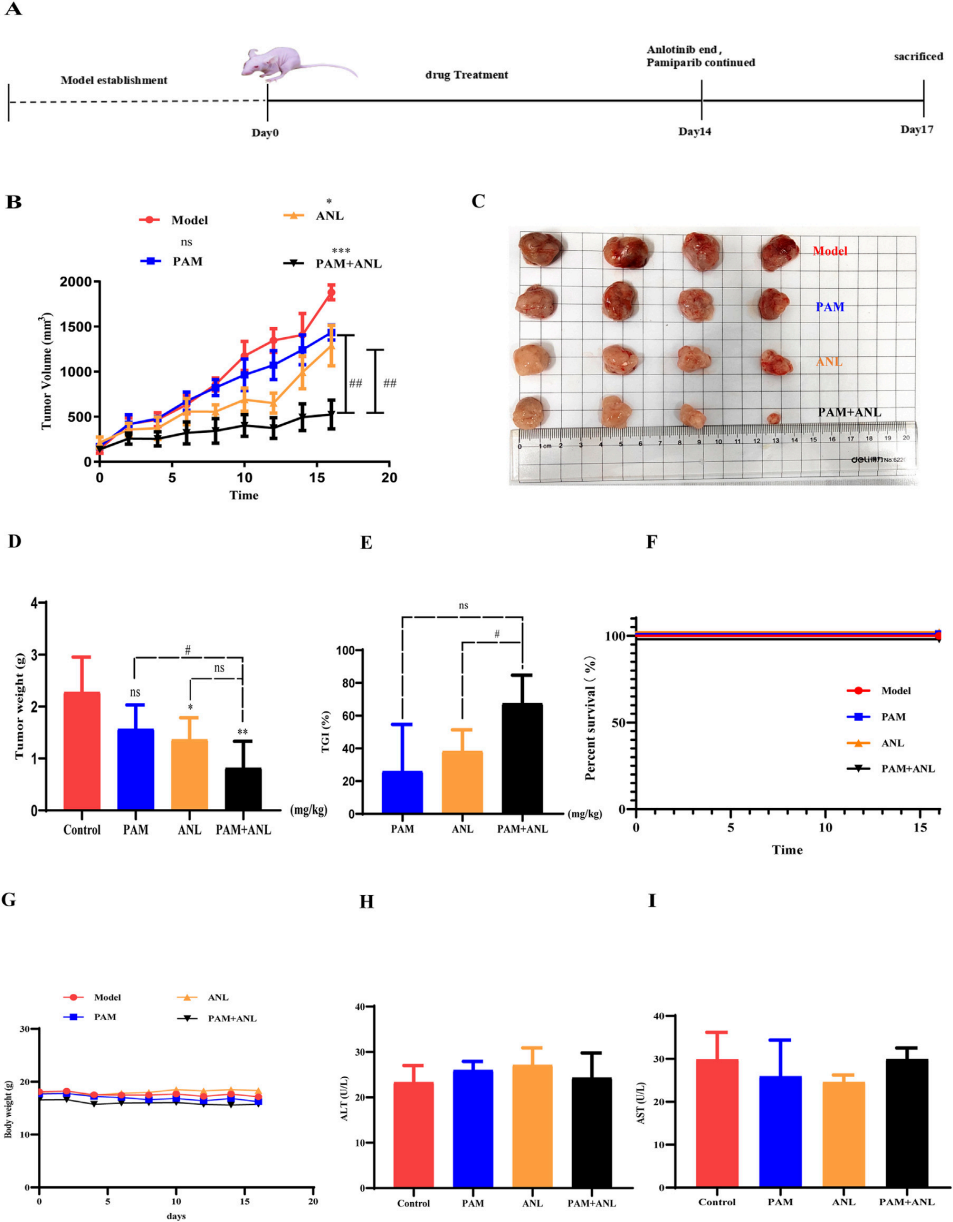
Antitumor activity of PAM and/or ANL in transplanted A2780 cells in nude mice. **(A)** Timeline illustrating the experimental schedule for nude mice. **(B)** Tumor volume changes in nude mice were recorded every 2 days during the whole treatment period (n = 4). **(C,D)** Tumor images and weight at the end of the experiment. **(E)** Tumor growth inhibition (TGI) quantification (n=4). **(F)** Survival analysis in the different treatment groups (n=4). **(G)** Body weight changes in the different treatment groups (n = 4). **(H,I)** Effect of drug administration on liver toxicity (n = 4). All data are representative of four independent experiments, and the values are expressed as the mean ± SEM. *p < 0.05, **p < 0.01, ***p < 0.001 compared with the model group. ##p < 0.01, PAM or ANL alone compared with their combination.

To evaluate the effect of *in vivo* combination therapy, we assessed the effects of PAM at a dose of 15 mg/kg and ANL at a dose of 1.5 mg/kg in a tumor-bearing mouse model (Figure 3A). Since there was no joint index model for the combination *in vivo*, we reduced the administration dose by 2/3, resulting in 10 mg/kg PAM combined with 1 mg/kg ANL. After 13 days of tumor growth and 16 days of administration, the PAM + ANL group showed some suppression of tumor volume compared to the model group (Figures 3B,C). The study results indicated that the combination treatment significantly controlled tumor growth *in vivo*. Tumors were weighed after the mice were sacrificed (Figure 3D). There was no statistically significant difference in tumor weight in the PAM group compared to the model group. The combination group showed the greatest statistical significance compared to the model group, indicating that the combination of the two drugs can inhibit tumor growth in mice *in vivo*. Tumor Growth Inhibition (TGI) values were calculated for each group: PAM monotherapy group: 26.08%, ANL monotherapy group: 38.32%, and the combination group: 67.54%. Among these, the TGI value of the combination group was significantly higher than that of either the PAM or ANL monotherapy group, indicating that the combination of the two drugs can effectively inhibit tumor growth (Figure 3E). In terms of survival, no mouse deaths were observed after 16 days of treatment (Figure 3F),There was no difference in the body weight of mice when the drugs were used alone or in combination, indicating the combination of drugs caused no toxicity (Figure 3G). Mouse serum was obtained after blood sampling from the brachial artery, and the levels of the liver toxicity indicators alanine aminotransferase (ALT) and aspartate aminotransferase (AST) were evaluated. PAM combined with ANL had almost no hepatotoxicity *in vivo* (Figures 3H,I). This demonstrated that the combination of these two drugs had good drug-forming properties *in vivo*.

### 3.6 *In vivo* and *in vitro* metabolomics analysis of PAM in combination with ANL

**FIGURE 4:**
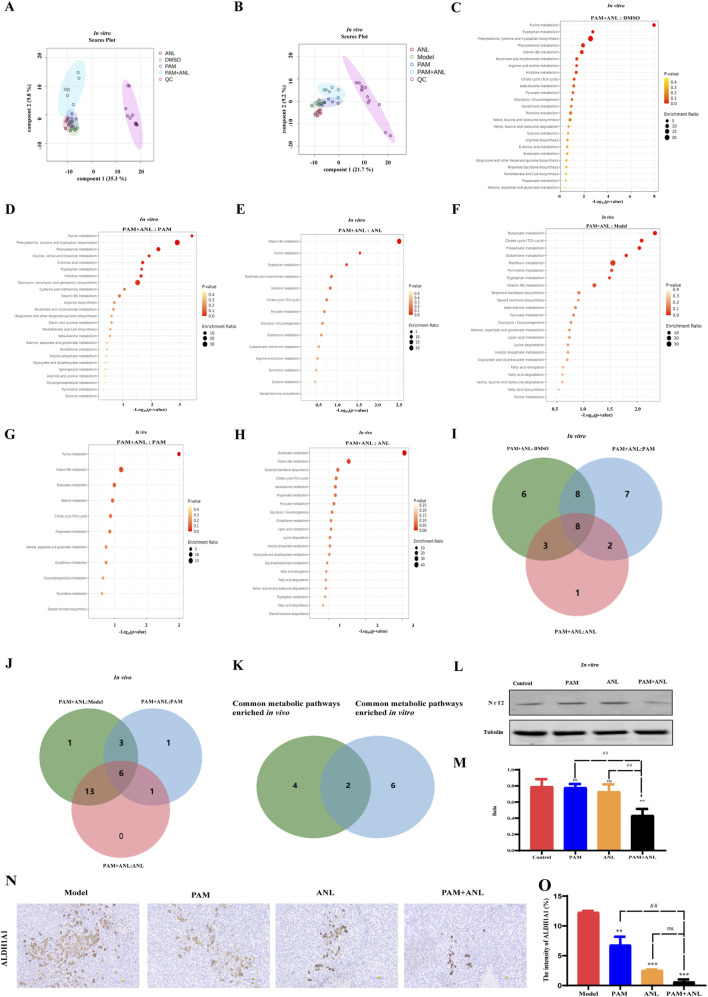
Metabolomics analysis of PAM and/or ANL. **(A,B)** PLS-DA plots of *in vitro* and *in vivo* experiments. **(C–E)** KEGG pathway enrichment analyses for the differential *in vitro* metabolites. **(F‐H)** KEGG pathway enrichment analyses for the differential *in vivo* metabolites. **(I)** Venny plots illustrated the overlap of *in vitro* metabolic pathway in PAM + ANL groups compare to other three groups. **(J)** Venny plots illustrated the overlap of *in vivo* metabolic pathway in PAM + ANL groups compared to other three groups. **(K)** Venny plots illustrated the overlap of common metabolic pathways that are enriched *in vitro* and *in vivo*. **(L,M)** Western blot of protein expression in A2780 cells treated with PAM(2 μM), ANL (2 μM), or PAM (2 μM)+ ANL (2 μM). **(N,O)** IHC of protein expression in A2780 cells treated with PAM(2 μM), ANL (2 μM), or PAM(2 μM) + ANL (2 μM). All data are representative of three independent experiments, and the values are expressed as the mean ± SD. **p < 0.01 compared with the model group. ^##^ *p <* 0.01, PAM or ANL alone compared with their combination.

After pre-processing the raw data, a partial least squares discriminant analysis (PLS-DA) was used for multiple comparisons to provide an overview of the metabolic profile. In cell samples, a trend toward separation was observed in the PLS-DA score plot when comparing PAM and ANL alone or in combination with the QC group (Figure 4A). In tissue samples, a trend of separation was also observed when comparing PAM and ANL alone or in combination with the QC group (Figure 4B). This indicates the instrument is stable enough for the next analysis. *In vivo*, the difference between PAM combined with ANL and MODEL, PAM, and ANL groups was analyzed using orthogonal partial least squares discriminant analysis (OPLS-DA). It was found that, when combined, the samples were well separated between the groups, suggesting differential metabolites between the groups. The same results were obtained in the *in vitro* analyses (Supplementary Figures S2, S3). Next, we analyzed the different metabolites between the *in vivo* and *in vitro* groups, setting the conditions as *p* < 0.05 and |log_2_FC| > 1. The difference between the metabolites in each group was significant (Supplementary Figures S4, S5). Subsequently, we performed KEGG pathway enrichment analyses for the differential metabolites between the groups separately, and the top 25 were selected for each group, as shown in (Figures 4C–H), where multiple metabolic pathways were significantly enriched *in vitro* and *in vivo*. We found that eight metabolic pathways—purine metabolism, tryptophan metabolism, vitamin B6 metabolism, nicotinate and nicotinamide metabolism, histidine metabolism, glutathione metabolism, arginine and proline metabolism, and tyrosine metabolism—are common *in vitro* (Figure 4I). *In vivo*, we performed the same analysis and the results showed that six metabolic pathways are common, including butanoate metabolism, citrate cycle (TCA cycle), propanoate metabolism, glutathione metabolism, vitamin B6 metabolism, and steroid hormone biosynthesis (Figure 4J). We took the intersection of common metabolic pathways *in vivo* and *in vitro* and found that glutathione metabolism, and vitamin B6 metabolism were common metabolic pathways, and we identified these two pathways as core metabolic pathways (Figure 4K). This suggests that new metabolic pathways are triggered when PAM + ANL is co-administered. To further verify, the protein expression of Nrf2, which closely related to glutathione metabolism (Cho et al., 2008), was evaluated. The results showed that PAM combined with ANL significantly downregulated the protein expression of Nrf2 (Figures 4L,M), which confirmed the combination of the two drugs is closely related to glutathione metabolism.

Accumulating evidence has demonstrated that Nrf2 can directly regulate the transcriptional expression of ALDH family molecules, and its high activity is a key driver for ALDH-positive tumor cells to maintain stem cell-like properties (Kim et al., 2018); Moreover, ALDH1A1’s catalytic activity and protein stability are highly dependent on reduced glutathione (GSH) to scavenge reactive oxygen species (ROS) generated during its metabolic reactions (Wang et al., 2017). We examined the expression of ALDH1A1, a core marker of cancer stem cells (CSCs). Immunohistochemical results showed that the expression intensity of ALDH1A1 was significantly decreased in the PAM + ANL group (Figures 4N,O), suggesting that the combination therapy can downregulate the expression of ALDH1A1, a core marker of CSCs, by inhibiting the Nrf2-mediated glutathione metabolic pathway, thereby suppressing tumor stemness.*3.7. In vivo and in vitro study of the mechanism of PAM in combination with ANL*.

**FIGURE 5:**
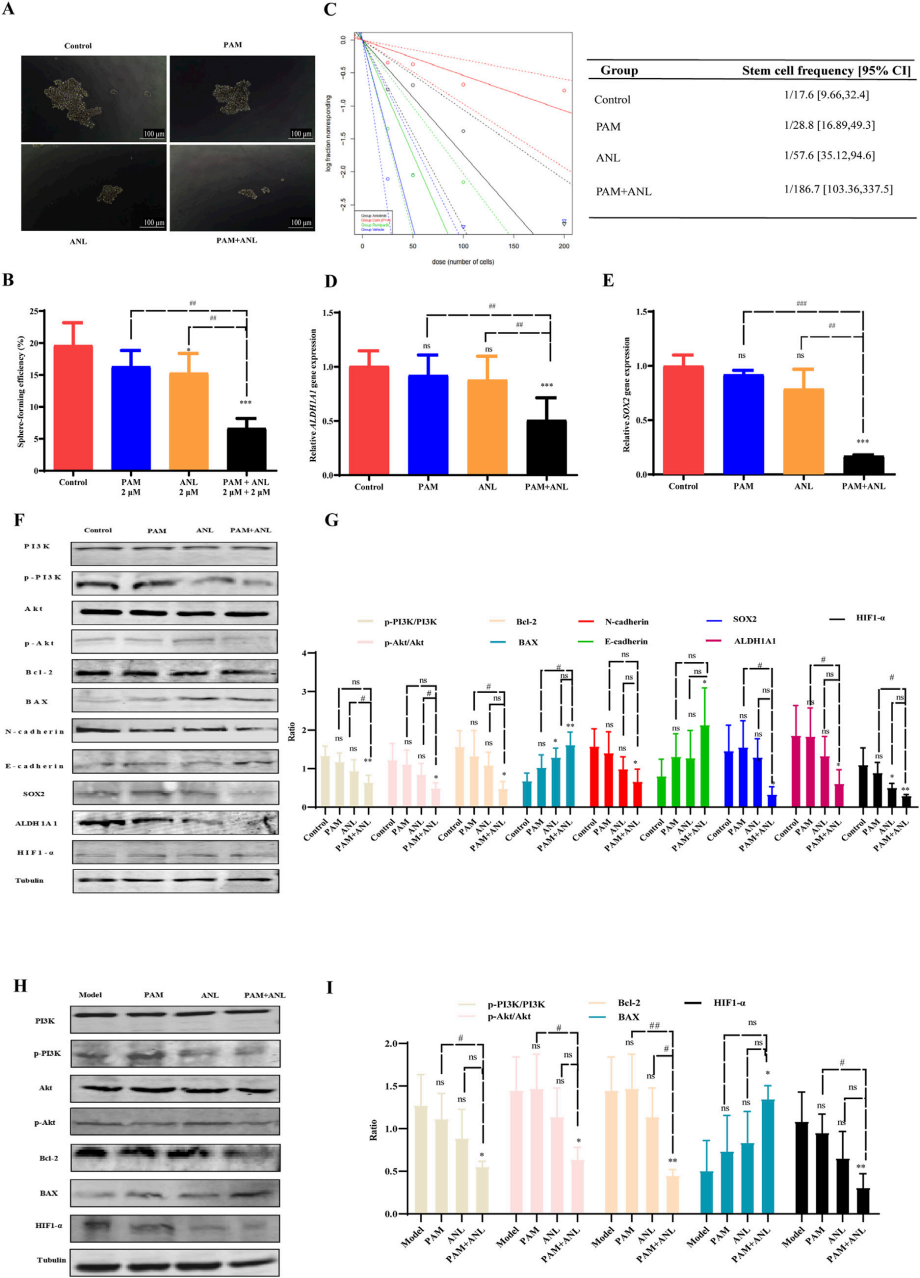
Mechanism of PAM combined with ANL analyzed *in vitro* and *in vivo*. **(A,B)** Tumor sphere assay results show the sphere-forming ability of stem cells treated with DMSO (control), PAM(0.5 μM), ANL (0.5 μM), and PAM + ANL (0.5 μM + 0.5 μM). **(C)** Limiting dilution experiments with blue solid and blue dashed lines for the control, green solid and green dashed lines for PAM (0.5 μM), black solid and black dashed lines for ANL (0.5 μM), and red solid and red dashed lines for the combination of two drugs (0.5 μM + 0.5 μM). The slope of the solid line represents the probability of tumor stem cell spheroid formation, and the slope of the dashed line represents the confidence interval for tumor stem cell spheroid formation. The above data are representative of ten independent experiments, and the values are expressed as the mean ± SD. **(D,E)** RT-qPCR experiments showing the effects of PAM(2 μM), ANL (2 μM)and two-drug combinations (2 μM + 2 μM)on tumor stem cell-related genes *SOX2* and *ALDH1A1*. **(F)** Western blot of protein expression in A2780 cells treated with PAM(2 μM), ANL (2 μM), or PAM(2 μM) + ANL (2 μM). **(G)** Quantitative analysis of protein levels in the *in vitro* experiment compared to that of the control. **(H)** Western blot of protein expression in tumor tissue treated with PAM(2 μM), ANL (2 μM), or PAM(2 μM) + ANL (2 μM). **(I)** Quantitative analysis of protein levels *in vivo*. Except for p-PI3K/PI3K and p-Akt/Akt, ratios were normalized to tubulin. *p < 0.05, **p < 0.01, ***p < 0.001 compared with the control (DMSO) or model group. All data are representative of three independent experiments, and the values are expressed as the mean ± SD. ^#^ *p <* 0.05, ^##^ *p <* 0.01, PAM or ANL alone compared with their combination.

Glutathione metabolism plays an integral role in maintaining the survival and stemness of CSCs (Jagust et al., 2020; Xiong et al., 2023), which are closely associated with cell migration and drug resistance (Safa, 2020). Subsequently, an in-depth verification was conducted. Cancer cells typically die due to a lack of nutrition, CSCs remain in suspension and grow into spheres. This demonstrates the stemness of the tumor cell group, which can be assessed by examining the number and size of these spheres. PAM combined with ANL significantly reduced the suspended clonal sphere formation rate (Figures 5A,B). The results indicated that the size of the spheres formed in the PAM + ANL group was significantly reduced compared to the control group. Furthermore, the combination of the two drugs had a statistically significant effect on inhibiting the ability of stem cells to form spheres compared with the monotherapy groups.

Sphere formation in stem cells was analyzed after drug treatment by diluting the cells to 200, 100, 50, and 25 cells/well. This study evaluated the effect of the combination of two drugs on the likelihood of stem cell sphere formation (Figure 5C). The figure displays the control group with blue solid and blue dashed lines, PAM with green solid and green dashed lines, ANL with black solid and black dashed lines, and the PAM + ANL combination with red solid and red dashed lines. The slope indicates the probability of success, with a steeper slope indicating a lower probability of success and a shallower slope indicating a higher probability of success. This result indicated that the combination of PAM + ANL significantly inhibited ovarian CSCs spherogenicity at extreme dilutions, as evidenced by a confidence interval of 1/169.7, which was lower than those in the single-drug and control groups.

We also confirmed the inhibitory effect of the drug combination on ovarian CSCs activity. qRT-PCR was used to evaluate the expression of *SOX2* and *ALDH1A1*, which are two genes associated with stem cells (Figures 5D,E). The combination group exhibited a statistically significant reduction in gene expression compared with both the control and monotherapy groups. This combination of drugs has the potential to significantly inhibit ovarian CSC-related gene expression and reduce drug resistance in ovarian cancer cells.

The PI3K/Akt signaling pathway plays a central role in the regulation of glutathione metabolism (Lien et al., 2016). The combination of PAM and ANL *in vitro* significantly downregulates the phosphorylation of PI3K and Akt, this indicates that the combination of the two drugs is related to the PI3K/Akt signaling pathway, resulting in a decrease in the expression of the downstream apoptosis-related protein Bcl-2 and an increase in the expression of BAX. Additionally, N-cadherin expression was reduced, whereas E-cadherin expression increased. These two cadherin proteins are associated with migration inhibition. Furthermore, the expression of stem cell-related proteins SOX2 and ALDH1A1 was also reduced. Moreover, the expression of HIF1-α, which is downstream of PI3K/Akt, was inhibited, indicating the inhibition of DNA HR repair and increased sensitivity to PARP inhibitors. Therefore, the combination of the two drugs inhibited growth, migration, EMT, and CSCs, and induced apoptosis of ovarian cancer through the PI3K/Akt signaling pathway *in vitro* (Figures 5F,G).

To verify the *in vivo* mechanism, we analyzed proteins of the tumor tissues of mice and conducted Western blotting experiments. PAM combined with ANL significantly downregulated the protein expression of p-PI3K, p-Akt, Bcl-2, and HIF1-α, and upregulated the protein expression of BAX. The *in vivo* results confirmed that the mechanism of action of the combination therapy is related to the PI3K/Akt pathway (Figures 5H,I).

## 4 Discussion

PARP inhibitors are used to treat patients with ovarian cancer, blocking the HR repair of cancer cells, and resulting in a “synthetic lethal effect.” However, prolonged PARP inhibitor use can lead to insensitivity for various reasons, irrespective of whether BRCA is mutated and whether the HRD pathway is normal (Kim et al., 2023). Here, PAM combined with ANL was identified through AI assisted methods (Figure 1F). We also found that a combination of low-dose PAM and low-dose ANL exhibits effective anticancer activity against ovarian cancer cells with minimal toxicity. A2780 cells were selected for this study because they are least sensitive to PAM and represent epithelial ovarian cancer cells, which constitute approximately 80% of patients with this type of cancer (Jelovac and Armstrong, 2011). The results indicated that PAM, ANL, or their combination inhibited cancer cell viability in a dose-dependent manner (Figure 2). However, the combination had a stronger inhibitory effect than a single agent. Therefore, we investigated the mechanisms triggered by this two-drug combination in ovarian cancer.

In the present study, we enriched the metabolic pathway, glutathione, by metabolomics analysis, and due to the dysregulation of the glutathione metabolic pathway, the expression of Nrf2 was reduced in ovarian cancer cells in the combination group compared with the control group and the single-agent group (Figures 4L,M). The expression of ALDH1A1 was also decreased in ovarian cancer tissues (Figures 4N,O). Meanwhile, upregulation of Nrf2 was observed in various tumors, including breast, ovarian, prostate, skin, lung, and pancreatic cancers (Gorrini et al., 2013; Kong and Chandel, 2018),The catalytic activity and protein stability of ALDH1A1 are highly dependent on reduced GSH (Wang et al., 2017), further supporting that the two-drug combination can affect the glutathione metabolic pathway. N-cadherin expression was reduced, and E-cadherin expression increased in combination-treated ovarian cancer cells compared to those in the control and single-agent groups, consistent with previous research (Haslehurst et al., 2012; Wang et al., 2014). Moreover, a growing number of studies have shown that EMT and CSCs share the same markers and properties, highlighting the significance of EMT in chemotherapy resistance in ovarian cancer (Shibue and Weinberg, 2017). Our results showed that in ovarian cancer cells, the inhibition of EMT biomarkers also inhibited CSC biomarkers, further demonstrating the close correlation between EMT and CSC.

The theory that CSCs provide a possible explanation for the recurrence of tumors after treatment and the development of drug resistance is highly relevant for ovarian cancer. Targeting CSCs is a promising approach that may enhance the effectiveness of chemotherapy and improve prognosis for patients with ovarian cancer (Deng et al., 2016). In this study, a combination of drugs was effective in inhibiting ovarian CSCs, as demonstrated by their reduced sphere-forming ability and stem cell marker expression after limiting dilution. These results suggest that drug combinations have the potential to overcome drug resistance in patients with ovarian cancer.

Mounting evidence indicates that the PI3K/Akt signaling pathway, EMT, and CSCs are significant factors in the progression, metastasis, and chemotherapy resistance of ovarian cancer (Deng et al., 2016). Therefore, inhibiting the PI3K/Akt signaling pathway, which inhibits EMT and CSC, through a combination of drugs can overcome drug resistance in ovarian cancer cells. Further research is required to fully understand the specific mechanisms by which this drug combination works. The combination of drugs exhibited superior inhibition of p-PI3K and p-Akt expression compared to monotherapy groups. Furthermore, the combination of drugs significantly inhibited EMT, CSC, and apoptotic protein biomarkers. Our study confirmed the relationship between EMT, CSC, and apoptosis, which are downstream of the PI3K/Akt signaling pathway (Figures 5F–I). Recent evidence demonstrates ANL’s capacity to reshape the tumor immune microenvironment and enhance anti-PD-1 efficacy through modulation of the VEGFR2/AKT/HIF-1α axis and TFRC-CXCL14 signaling (Song et al., 2024). Our data show that PAM + ANL combination downregulates HIF1-α expression via the PI3K/Akt pathway (Figures 5F–I), and this new evidence extends the understanding of ANL’s regulation of AKT-HIF1α axis to immunomodulatory effects, beyond direct inhibition of tumor cell proliferation and stemness. It also highlights the translational relevance of ANL-based combination strategies across tumor types, providing additional support for the clinical potential of the PAM + ANL regimen in ovarian cancer, especially in scenarios where immunotherapeutic integration may be considered. However, we did not conduct specific experiments focusing on the mechanism by which PAM and ANL overcome drug resistance and inhibit ovarian CSCs. Therefore, we will focus on the synergy between PAM and ANL that contributes to enhanced efficacy, particularly in the context of paclitaxel or platinum-resistant cells. The drug combination studied offers a novel insight into the pathogenesis of drug resistance, which can help develop strategies to avoid drug resistance in ovarian cancer. These findings provide a valuable reference for future clinical practice.

## 5 Conclusion

In conclusion, the combination of PAM and ANL inhibited the proliferation, migration, and stem cell activity of A2780 cells through the inhibition of p-PI3K and p-Akt, which in turn inhibited Nrf2, thereby affecting metabolism. Downregulation of the anti-apoptotic protein Bcl-2 and upregulation of BAX expression were observed. The combination was found to have a greater ability to inhibit cancer cell proliferation and promote apoptosis than either drug alone. The drug combination downregulated the EMT-related protein N-cadherin and upregulated E-cadherin, which is indicative of impaired cell migration. In addition, the stemness-related proteins SOX2 and ALDH1A1 were downregulated, indicating reduced ovarian stem cell activity. These effects contributed to reduced drug resistance and enhanced antitumor effects. The combination of these two drugs provides an innovative approach to drug administration for patients with drug-resistant ovarian cancer. In addition, EMT and CSC are key factors in chemoresistance in ovarian cancer patients, and their biomarkers indicate a poor prognosis. Targeted inhibition of EMT and CSC has the potential to improve the prognosis of ovarian cancer patients and provide a novel solution to the poor prognosis of these patients.

## Data Availability

The raw data supporting the conclusions of this article will be made available by the authors, without undue reservation.
