# Supplementary material for: Drug combination discovery assisted by AI and untargeted metabolomics: pamiparib and anlotinib synergistic potentiation for ovarian cancer treatment
Source: Front Pharmacol. 2025 Nov 26;16:1702014. doi: 10.3389/fphar.2025.1702014 (PMC12689557; doi:10.3389/fphar.2025.1702014)
Supplement: Supplementary file 1 [file Supplementaryfile1.docx]

Figure S1. The cytotoxicity of PAM, ANL, and their combination on A2780 cells. **p <* 0.05, ****p <* 0.001 compared with the control (DMSO) group. All data are representative of three independent experiments, and the values are expressed as the mean ± SD. ^###^*p <* 0.001 PAM or ANL alone compared with their combination.


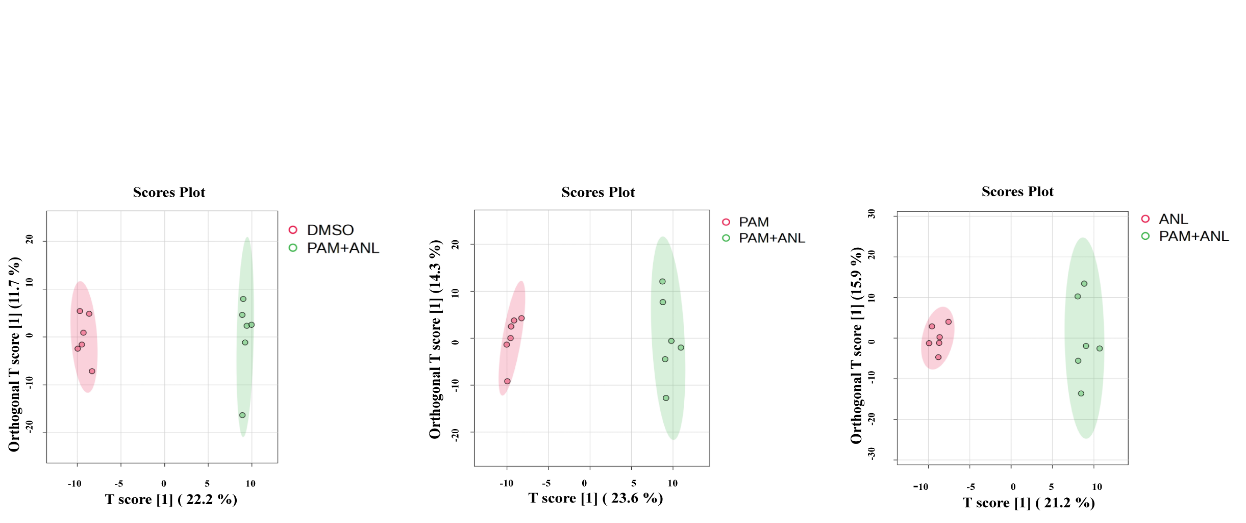


Figure S2. Orthogonal Partial Least Squares Discriminant Analysis (OPLS-DA) Differences between the PAM combined with ANL group and the DMSO, PAM, and ANL groups *in vitro.*


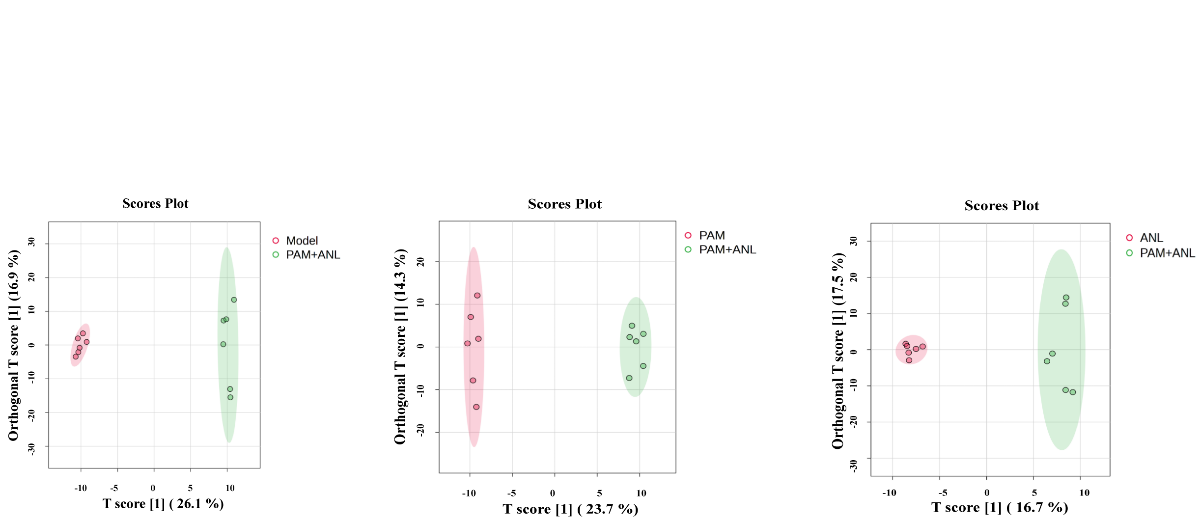


Figure S3. Orthogonal Partial Least Squares Discriminant Analysis (OPLS-DA) Differences between the PAM combined with ANL group and the Model, PAM, and ANL groups *in vivo*.


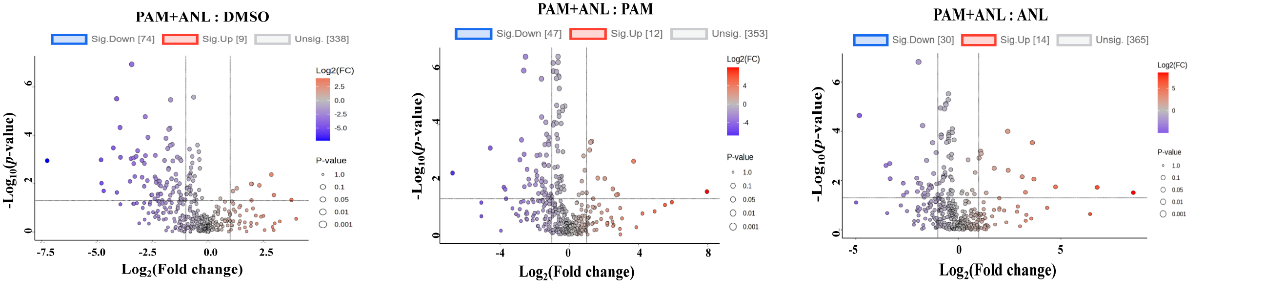


Figure S4. Differences in the expression of PAM+ANL :DMSO, PAM+ANL : PAM and PAM+ANL : ANL metabolites were analysed *in vitro* by volcano plots.


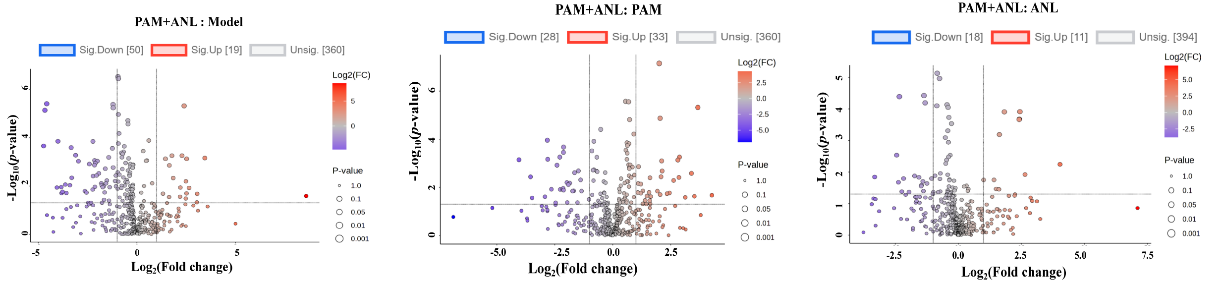


Figure S5. Differences in the expression of PAM+ANL : Model, PAM+ANL : PAM and PAM+ANL : ANL metabolites were analysed *in vivo* by volcano plots.

Table S1. The screening dosing ratios and CI Values of PAM, ANL, and their combination on A2780 cell.

| Combinations | | CI | Combinations | | CI |
| --- | --- | --- | --- | --- | --- |
| Dose PAM: μM | Dose ANL: μM |  | Dose PAM: μM | Dose ANL: μM |  |
| 32 | 8 | 0.05 | 8 | 1 | 0.61 |
| 32 | 4 | 0.20 | 8 | 0.5 | 0.58 |
| 32 | 2 | 0.22 | 8 | 0.25 | 0.65 |
| 32 | 1 | 0.19 | 4 | 8 | 0.63 |
| 32 | 0.5 | 0.21 | 4 | 4 | 0.69 |
| 32 | 0.25 | 0.24 | 4 | 2 | 0.51 |
| 16 | 8 | 1.42 | 4 | 1 | 0.48 |
| 16 | 4 | 0.88 | 4 | 0.5 | 0.45 |
| 16 | 2 | 0.65 | 4 | 0.25 | 0.62 |
| 16 | 1 | 0.70 | 2 | 8 | 0.74 |
| 16 | 0.5 | 0.82 | 2 | 4 | 0.87 |
| 16 | 0.25 | 1.04 | 2 | 2 | 0.57 |
| 8 | 8 | 1.01 | 2 | 1 | 0.39 |
| 8 | 4 | 0.87 | 2 | 0.5 | 0.50 |
| 8 | 2 | 0.69 | 2 | 0.25 | 1.00 |
